# Global Research Status and Trends in Venous Thromboembolism After Hip or Knee Arthroplasty From 1990 to 2021: A Bibliometric Analysis

**DOI:** 10.3389/fmed.2022.837163

**Published:** 2022-04-07

**Authors:** Wei Song, Tao Ma, Qianyue Cheng, Pengfei Wen, Jiayuan Wu, Linjie Hao, Binfei Zhang, Yakang Wang, Qiuyuan Wang, Yumin Zhang

**Affiliations:** ^1^Department of Joint Surgery, Honghui Hospital, Xi'an Jiaotong University, Shaanxi, China; ^2^Xi'an Medical University, Shaanxi, China; ^3^Department of Spine Surgery, Honghui Hospital, Shaanxi, China; ^4^Department of Orthopedics, China-Japan Friendship Hospital, Beijing, China

**Keywords:** venous thromboembolism (VTE), hip arthroplasty, knee arthroplasty, bibliometric analysis, hotspots, research trends

## Abstract

**Background:**

Venous thromboembolism (VTE) after hip or knee arthroplasty has attracted increasing attention over the past few decades. However, there is no bibliometric report on the publications in this field. The purpose of this study was to analyze the global research status, hotspots, and trends in VTE after arthroplasty.

**Methods:**

All articles about VTE research after hip or knee arthroplasty from 1990 to 2021 were retrieved from the Web of Science Core Collection database. The information of each article including citation, title, author, journal, country, institution, keywords, and level of evidence was extracted for bibliometric analysis.

**Results:**

A total of 1,245 original articles from 53 countries and 603 institutions were retrieved. The USA contributed most with 457 articles, followed by England and Canada. McMaster University in Canada was the leading institution for publications. The journals with the highest output and citation were the Journal of Arthroplasty and the Thrombosis and Haemostasis, respectively. The median number of citations was significantly different among the levels of evidence (*F* = 128.957, *P* < 0.001). The research hotspots switched from VTE diagnosis and heparin to factor Xa inhibitors (fondaparinux, rivaroxaban, apixaban) and direct thrombin inhibitors (dabigatran etexilate, ximelagatran), and finally to aspirin, risk factor studies, which can be observed from the keyword analysis and co-cited reference cluster analysis.

**Conclusions:**

This study observed an increasing trend of research articles on VTE after arthroplasty. Publications with higher levels of evidence gained further popularity among researchers and orthopedic surgeons. Additionally, individualized VTE prevention and the development of new, safe, effective, and inexpensive oral agents would be emerging trends in the future.

## Introduction

Postoperative venous thromboembolism (VTE) is a major cause of morbidity, mortality, and healthcare costs in patients undergoing knee or hip arthroplasty (1). VTE includes deep vein thrombosis (DVT) and pulmonary embolism (PE). The incidence of any (including asymptomatic) VTE in patients undergoing major orthopedic procedures without prophylaxis is ~40–60% (2). Up to 70% of VTE cases may be asymptomatic, about 6% of DVT and 12% of PE cases die within 1 month after diagnosis (3). To reduce the incidence of VTE, appropriate management strategies and guidelines were developed, such as the Caprini score (4), American Academy of Orthopaedic Surgeons (AAOS) guidelines (5), and American College of Chest Physicians (ACCP) guidelines (6, 7). Recently, the incidence of symptomatic VTE is ~0.45–5.30% after total knee arthroplasty (TKA) and 0.24–1.60% after total hip arthroplasty (THA) (8, 9).

The three physiological factors that may contribute to VTE are known as Virchow's triad, including venous stasis, endothelial injury, and hypercoagulable state. Two or more factors are typically necessary for the development of VTE (10). The VTE event is believed to be initiated during surgeries as a result of direct or indirect vessel wall trauma and intimal damage that induce a hypercoagulable state (11). In clinical studies, several risk factors for VTE have been determined, including but not limited to comorbidities, advanced age, cancer, prolonged immobilization, bilateral procedures, and longer operative time (12). With the rapidly increasing number of joint replacement procedures annually (13), VTE has attracted increasing attention, and publications on VTE research have grown dramatically over the past few decades. This causes challenges in fully understanding, accessing, and identifying relevant information in the field. To facilitate clinicians in assessing the overall success of interventions and to guide future research on VTE, it is necessary to shed light on research trends, hot spots, and high-impact articles, institutions, and authors in the field.

Bibliometric analysis is a novel and powerful method to estimate the characteristics and quality of articles or review an extensive field of knowledge, especially when confronted with an increasing number of publications (14). Such a tool can evaluate the scientific value of articles, show the current status, reveal research hotspots, and predict the trends of a specific area (15, 16). Many fields have published bibliometric analyses in their specialty, such as urological surgery (17), pharmacology (16), orthopedic surgery (15, 18, 19). However, to the best of our knowledge, there is still a lack of bibliometric analysis on VTE after lower extremity joint replacement. This study aimed to identify research trends and hotspots for VTE after hip and knee arthroplasty via integrative analysis of articles published worldwide.

## Methods

### Data Sources and Search Strategy

Relevant literature was searched from the Web of Science Core Collection (WOSCC) database on August 23, 2021. The search work was completed within 1 day to avoid biases caused by database updates (update: August 22, 2021). The search strategy was shown as follows, TS = [thromb^*^ and (((hip or knee) and (arthroplasty or replacement)) or hemiarthroplasty)], without any language restrictions, and the time-span was set as 1990–2021. The asterisk was used to extend the search, such as “thromb^*^” will search for thromboembolism, thrombosis, and thromboprophylaxi. Only original articles were reserved and all other categories were excluded.

### Data Selection

**Figure 1:**
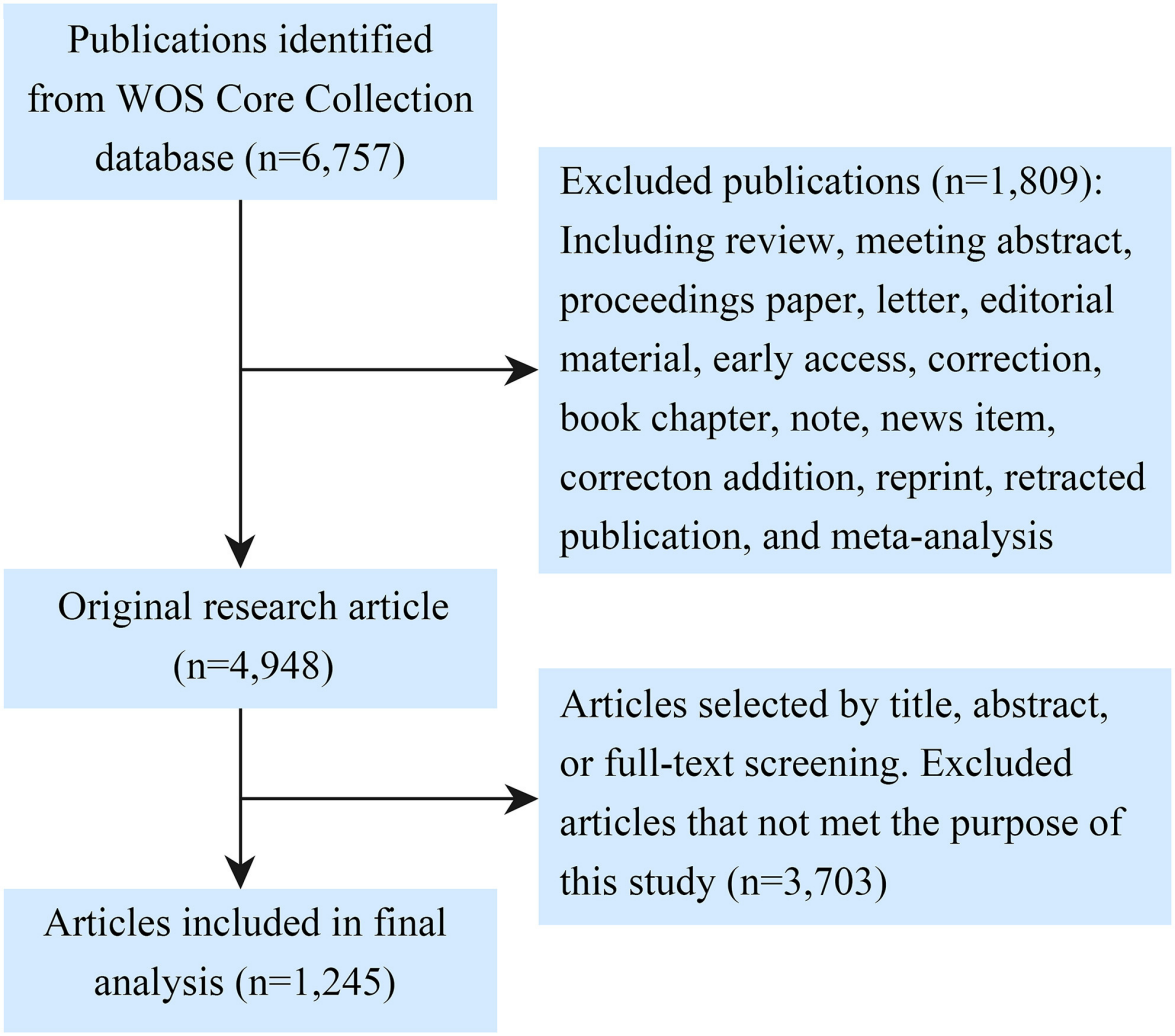
Flowchart of the identification of relevant articles.

Articles were screened by two authors independently. Unrelated studies were removed and disagreements were resolved by mutual agreement. Finally, 1,245 articles were then assessed further. The screening process is shown in Figure 1. The following data of each article were collected: publication years, journals, impact factors (IF) obtained from the Journal Citation Report (JCR 2020), titles, authors, countries, institutions, references, keywords, citation counts, citations per year (total citations/the number of years since publication), and levels of evidence evaluated according to guidelines of the Journal of Bone and Joint Surgery (20).

### Data Analysis

The data were imported into Microsoft Excel 2016 (Microsoft Corp., Redmond, WA, USA), the online analysis platform of literature metrology (https://bibliometric.com), and CiteSpace 5.7.R3 (Drexel University, Philadelphia, PA, USA) for analysis. The online analysis platform was utilized to visualize the analysis of the cooperation network of countries. CiteSpace was used to visualize analyses of the co-occurrence keywords and the cooperation network of institutions and authors, and to further construct a timeline view of co-cited references by which we could clarify the rise and period of certain clustering fields (14). Burst detection was used to identify the keyword bursts which indicate frontier concepts and emerging trends that have drawn the attention of peer investigators (21). As for visual graphs, the colors of the circular node and connections represent the period in which the articles were published. The overall time-span from 1990 to 2021, with 4 years per slice, was divided into 8 different time slices corresponding to 8 different colors.

Graphs were made by OriginPro 9.1 (OriginLab Corp., Northampton, Massachusetts, USA). The analyses were performed by Microsoft Excel, and IBM SPSS 22.0 (IBM Corp., Armonk, NY, USA). Non-normal distributed data were analyzed by the Kruskal-Wallis test. The results were presented as the median (the first and third quartiles). Correlation between variables was tested by Kendall's tau-b test. *P* < 0.05 was defined as statistically significant.

## Results

### Publication and Citation

**Figure 2:**
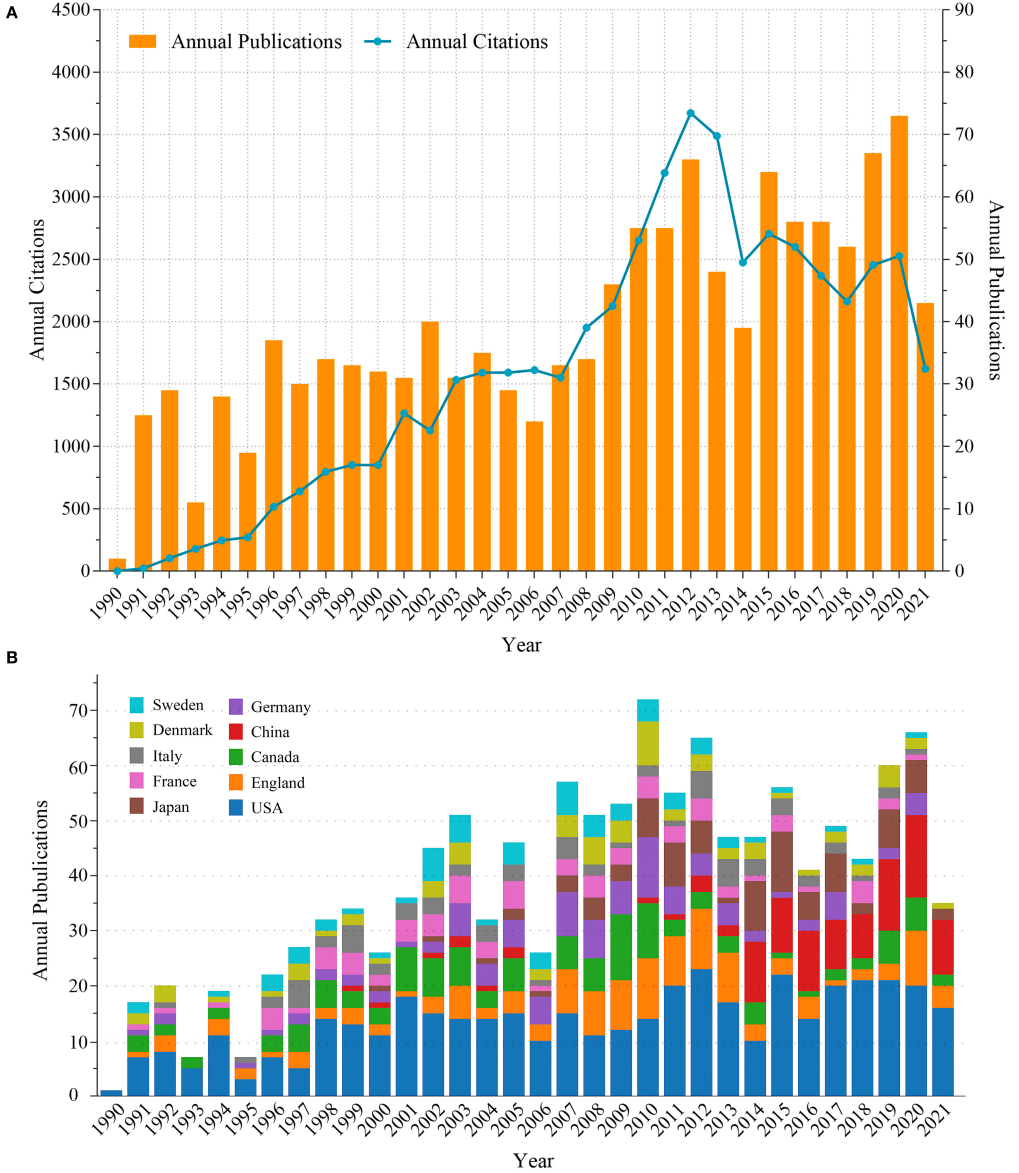
Trends in annual citations and publications **(A)** and trends in annual article counts in top 10 productive countries **(B)** on VTE after hip or knee arthroplasty.

A total of 1,245 articles on VTE after hip and knee arthroplasty were collected from the WoSCC database between 1990 and 2021. Most of these articles were in English (95.3%), followed by German (2.1%) and French (1.4%). From 1990 to 2012, the number of annual articles showed an increasing trend, with the largest number of 73 articles published in 2020, as shown in Figure 2A. More than half of the articles were published in the last decade. When it comes to the citation number, the total number of citations of these articles was 50,743, without self-citations was 41,800. It could be seen from the chart that the annual citations showed an increasing trend and peaked in 2012, followed by a downward trend (Figure 2A).

### Country and Institution

**Figure 3:**
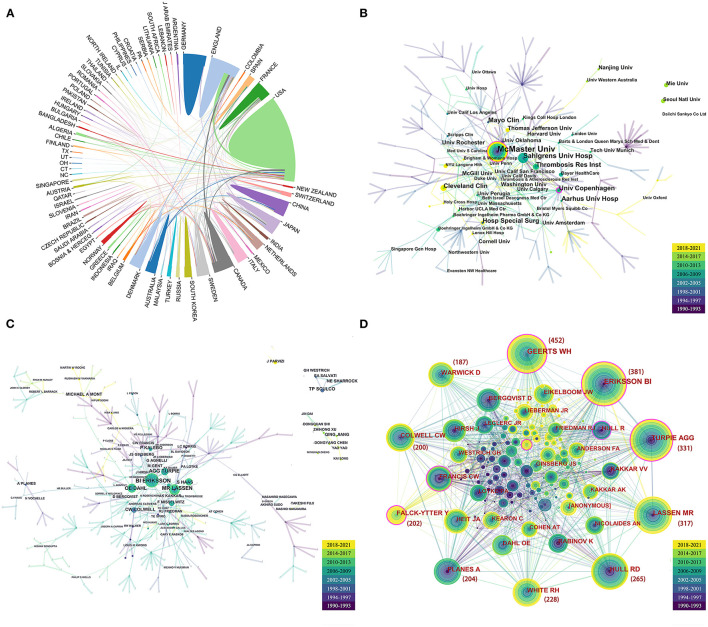
Cooperation network among countries **(A)**, institutions **(B)**, and authors **(C)** and the graph of co-cited author analysis **(D)**. The nodes in the graph represent institutions or authors, and lines between the nodes represent the collaborative relationships and co-cited relationships. The various colors in the nodes represent the different years, and the larger the node area, the greater the number of publications and co-citations. The nodes with purple rings represent high centrality and are considered to be pivotal points in the study.

Articles originated from 53 countries. The publications in the top 10 productive countries were demonstrated by years (Figure 2B) and shown in Table 1. The USA was the most productive country in this field, with 457 articles (36.7%), followed by England, Canada, China, Germany, Japan, France, Denmark, Italy, and Sweden. It was apparent from Figure 2B that the production of articles from China has increased rapidly since 2014. However, no clear increasing trend was observed in other countries. The USA has consistently maintained first place in the number of annual articles on VTE after arthroplasty. Furthermore, the cooperation network of countries showed that the collaborations among the USA, Canada, and England occurred frequently, followed by that between England and Denmark, and the USA and Australia (Figure 3A).

**Table 1:** List of top 10 countries according to the total number of publications.

**Rank**	**Country**	**Number of articles**	**Total citation**	**Average citation**
1	USA	457	14,199	31.07
2	England	132	4,060	30.76
3	Canada	129	8,223	63.74
4	China	102	612	6.00
5	Germany	91	2,586	28.42
6	Japan	87	1,349	15.51
7	France	78	1,772	22.72
8	Denmark	71	4,489	63.23
9	Italy	70	1,394	19.91
10	Sweden	65	5,556	85.48

A total of 603 institutions have participated in the study of VTE after arthroplasty. Half of the top 10 most prolific institutions were located in the USA, as shown in Table 2. The most prolific institution was the McMaster University from Canada with 61 publications, followed by Sahlgrens University (*n* = 33), University of Copenhagen (*n* = 27), and Mayo Clinic (*n* = 27). The visualized analysis could reveal a network of correlations among institutions (15). The network maps among institutions showed a low density (density = 0.0031) (Figure 3B), indicating that research teams are relatively independent, thereby necessitating further collaborations. McMaster University and the University of Copenhagen had high centrality (0.13 and 0.21, respectively), which suggested that these institutions have a great influence on VTE research.

**Table 2:** List of top 10 contributing institutions and their countries.

**Rank**	**Institutions**	**Articles**	**Total citations**	**Average citations**	**Country**
1	McMaster University	61	8,214	134.66	Canada
2	Sahlgrens University	33	6,994	211.94	Sweden
3	University of Copenhagen	27	4,026	149.11	Denmark
4	Mayo Clinic	27	1,523	56.41	USA
5	Thrombosis Research Institute	25	5,731	229.24	England
6	Aarhus University Hospital	25	3,335	133.40	Denmark
7	Cleveland Clinic	24	303	12.63	USA
8	Hospital for Special Surgery	24	806	33.58	USA
9	Thomas Jefferson University	18	642	35.67	USA
10	University of Rochester	17	2,552	150.12	USA

### Author and Co-cited Author

These articles were contributed by at least 1,321 authors. The researchers and their collaborations were shown in Figure 3C. Table 3 illustrated the top 10 productive authors, i.e., those who authored at least 19 articles. The person leading the ranking was Eriksson BI (Sahlgrens University, Sweden) with 42 articles totalizing 7,915 citations and had the closest cooperation with other authors. Lassen MR from Nordsjaellands Hospital, the second most prolific author, was ranked first in terms of citations. Figure 3D showed the co-cited author network. The authors with the top five co-cited counts were Geerts WH (452), Eriksson BI (381), Turpie AGG (331), Lassen MR (317), and Hull RD (265).

**Table 3:** Top 10 most prolific authors on VTE research after hip or knee arthroplasty.

**Rank**	**Author**	**Article** **counts**	**Total** **citation**	**Average** **citation**	**Institution**	**Country**
1	Eriksson, BI	42	7,915	188.45	Sahlgrens University	Sweden
2	Lassen, MR	39	8,303	212.90	Nordsjaellands Hospital	Denmark
3	Dahl, OE	32	6,274	196.06	Oslo University	Norway
4	Turpie, AGG	30	5,305	176.83	Hamilton General Hospital	Canada
5	Sculco, TP	23	844	36.70	Hospital for Special Surgery	USA
6	Kalebo, P	19	3,112	163.79	University of Gothenburg	Sweden
7	Colwell, CW	18	3,689	204.94	Scripps Clinic Medical Group	USA
8	Haas, SB	17	2,917	171.59	Hospital for Special Surgery	USA
9	Borris, LC	17	3,098	182.24	Aalborg Hospital	Denmark
10	Sharrock, NE	15	556	37.07	Hospital for Special Surgery	USA

### Journal

During the last three decades, a total of 332 academic journals published research articles on VTE after arthroplasty. The characteristics of the top 10 journals were shown in Table 4, among which the articles were predominantly published in Journal of Arthroplasty (*n* = 112), and followed by Thrombosis and Haemostasis (*n* = 68), Journal of Bone and Joint Surgery-British Volume (*n* = 52), Journal of Bone and Joint Surgery-American Volume (*n* = 48). Most of the publishers are situated in the USA, followed by England and Germany. In addition, half of the journals in the top 10 list reached an average number of citations >50. Thrombosis and Haemostasis ranked first in total citations, while Archives of Internal Medicine had achieved the highest impact factor and average citations with 19 articles on the list.

**Table 4:** List of top 10 journals with the most publications and their impact factors.

**Rank**	**Journal**	**Country**	**Number of articles**	**Total citation**	**Average citation**	**IF (2020)**
1	Journal of arthroplasty	USA	112	2,282	20.38	4.757
2	Thrombosis and haemostasis	Germany	68	5,290	77.7	5.249
3	Journal of bone and joint surgery-British volume^†^	England	52	3,109	59.79	5.082
4	Journal of bone and joint surgery-American volume	USA	48	3,509	73.10	5.284
5	Thrombosis research	England	47	1,219	25.94	3.944
6	Clinical orthopedics and related research	USA	42	1,253	29.83	4.176
7	Journal of thrombosis and haemostasis	USA	37	2,816	76.11	5.824
8	Orthopedics	USA	29	343	11.83	1.390
9	Clinical and applied thrombosis-hemostasis	USA	23	441	19.17	2.389
10	Archives of internal medicine^‡^	USA	19	2,694	141.79	21.873

† *In September 2011, JBJS (Am) and JBJS (Br) reached a joint agreement on future, independent operations. JBJS (Br) relaunched as Bone & Joint Journal in 2013. The impact factor (IF) of this Journal was 5.082 in 2020*.

‡ *Archives of Internal Medicine relaunched as JAMA Internal Medicine in 2013. The IF of this Journal was 21.873 in 2020*.

### Level of Evidence

**Figure 4:**
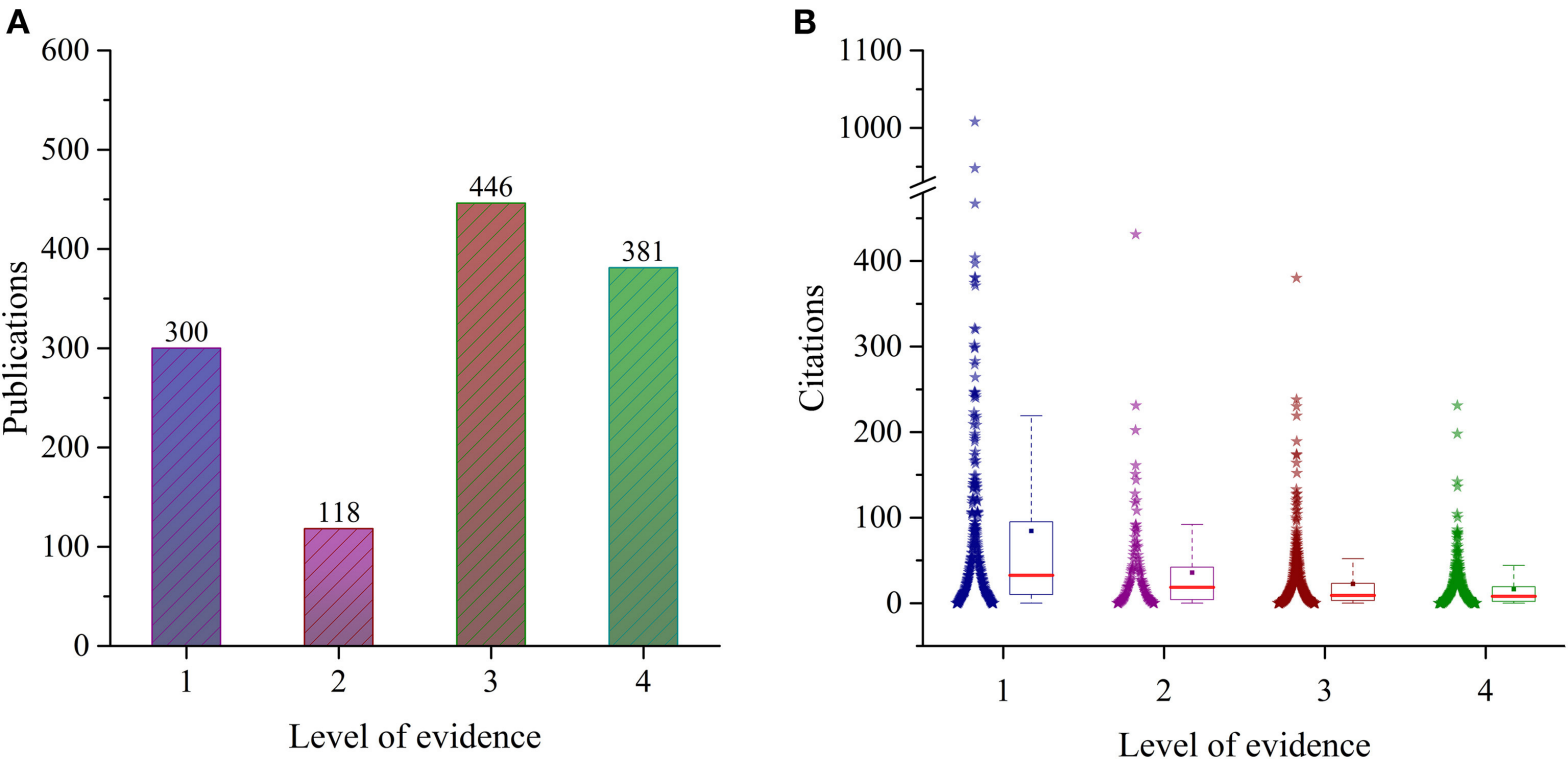
Number of publications **(A)** and citations (data distribution and interquartile range) **(B)** in each level of evidence.

There were 300 articles with level I evidence that were cited a median of 32.5 (10, 95) times, 118 articles with level II evidence that were cited a median of 18.5 (4, 42) times, 446 articles with level III evidence that were cited a median of 9 (3, 22) times and 446 articles with level IV evidence that were cited a median of 8 (2, 19) times (Figure 4). The Kruskal-Wallis test showed that the median number of citations was significantly different among the levels of evidence (*F* = 128.957, *P* < 0.001). There was a low positive correlation between the level of evidence and citations by Kendall‘s tau-b test (*r* = 0.232, *P* < 0.001).

### Top 10 Cited Articles and Co-cited References Clusters

**Figure 5:**
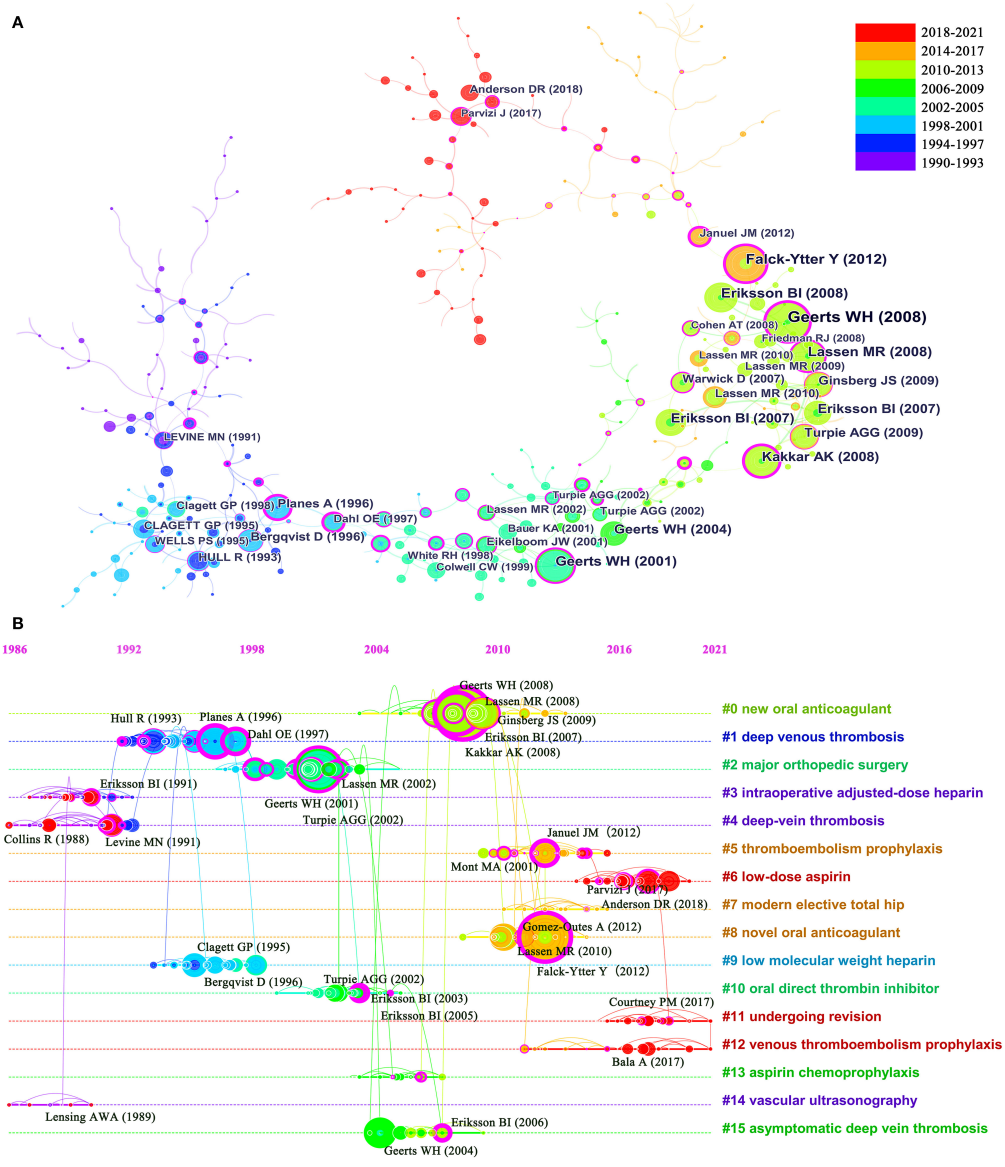
The co-cited reference knowledge map **(A)** and the timeline graph of co-cited reference clustering **(B)**.

Table 5 showed the details of the top 10 cited articles with citations ranging from 446 to 1,008. Four articles of these were published in The New England Journal of Medicine (IF = 91.24), and four in The Lancet (IF = 79.32). The main topics can be obtained by analyzing the co-cited references. Figure 5A showed the co-cited reference knowledge map. Furthermore, by analyzing the timeline graph generated by clustering co-cited references (Figure 5B), we can track the changes of researchers' focus on this field over time. The network of co-cited references was divided into 16 co-citation clusters. The labels of each cluster were extracted using a log-likelihood ratio. The silhouettes of clusters were all higher than 0.7, ranging from 0.897 (#11) to 1 (#6), which indicated the high consistency of clustered members. The ranking of the clusters was determined by the number of cited articles in the cluster.

**Table 5:** Top 10 high-cited articles related to venous thrombosis after hip or knee arthroplasty.

**Rank**	**Author**	**Year**	**Article**	**Journal**	**Citation**
1	Eriksson, BI	2008	Rivaroxaban vs. enoxaparin for thromboprophylaxis after hip arthroplasty	New England Journal of Medicine	1,008
2	Lassen, MR	2008	Rivaroxaban vs. enoxaparin for thromboprophylaxis after total knee arthroplasty	New England Journal of Medicine	948
3	Eriksson, BI	2007	Dabigatran etexilate vs. enoxaparin for prevention of venous thromboembolism after total hip replacement: a randomized, double-blind, non-inferiority trial	Lancet	834
4	Kakkar, AK	2008	Extended duration rivaroxaban vs. short-term enoxaparin for the prevention of venous thromboembolism after total hip arthroplasty: a double-blind, randomized controlled trial	Lancet	785
5	Turpie, AGG	2009	Rivaroxaban vs. enoxaparin for thromboprophylaxis after total knee arthroplasty (RECORD4): a randomized trial	Lancet	696
6	Lassen, MR	2010	Apixaban vs. enoxaparin for thromboprophylaxis after knee replacement (ADVANCE-2): a randomized double-blind trial	Lancet	553
7	Lassen, MR	2010	Apixaban vs. Enoxaparin for Thromboprophylaxis after Hip Replacement.	New England Journal of Medicine	514
8	Lassen, MR	2009	Apixaban or Enoxaparin for Thromboprophylaxis after Knee Replacement	New England Journal of Medicine	508
9	Ginsberg, JS	2009	Oral Thrombin Inhibitor Dabigatran Etexilate vs North American Enoxaparin Regimen for Prevention of Venous Thromboembolism After Knee Arthroplasty Surgery	Journal of Arthroplasty	490
10	White, RH	1998	Incidence and time course of thromboembolic outcomes following total hip or knee arthroplasty	Archives of Internal Medicine	446

### Keywords and Research Interest

**Figure 6:**
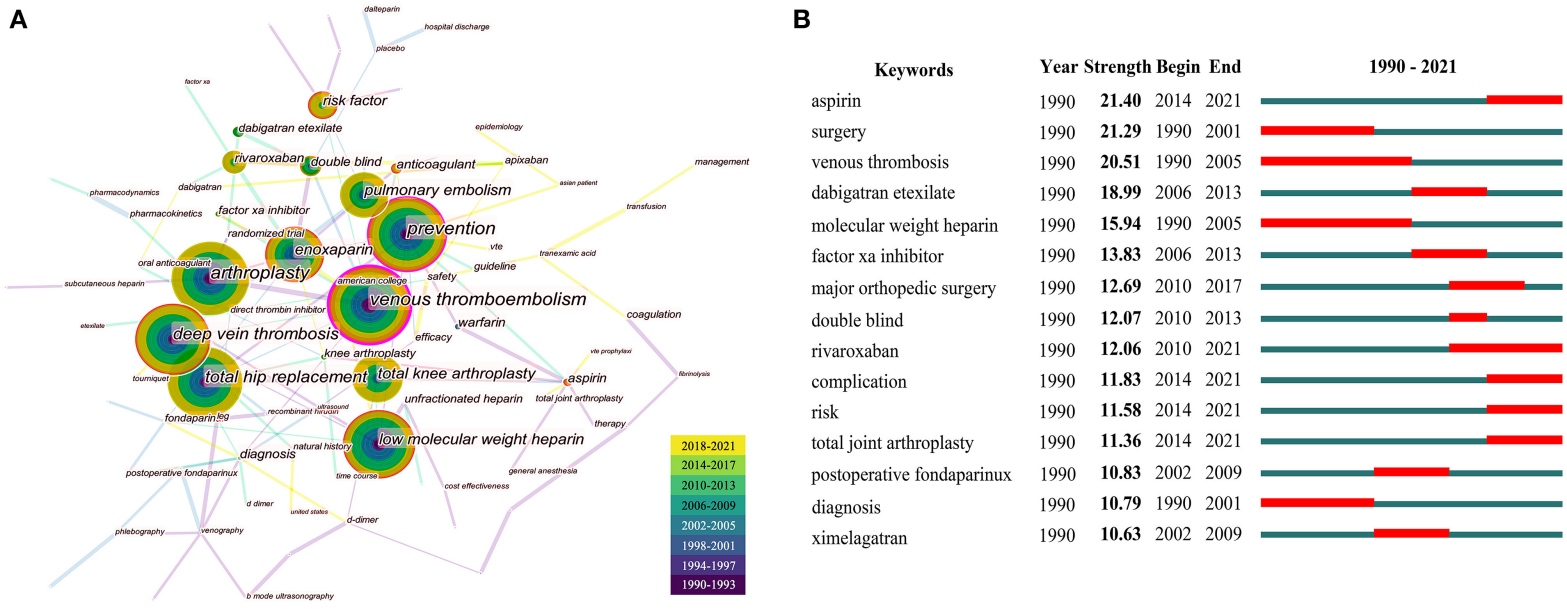
Co-occurrence map of keywords **(A)** and top 15 keywords with the strongest citation bursts **(B)**.

Figure 6A showed the co-occurrence keywords knowledge graph. The same meaning words were merged and meaningless words were excluded by CiteSpace. The keywords like “venous thromboembolism,” “arthroplasty,” “prevention,” “deep vein thrombosis,” “low molecular weight heparin,” “enoxaparin,” “pulmonary embolism,” and “risk factor” were the eight keywords used more frequently than 300 in the documents analyzed. A keyword burst was detected based on the analysis of all articles (Figure 6B). The timeline was described as a blue line indicating the beginning year and the ending year, while the period of a burst was marked as a red line indicating the time-span of a citation burst. Keywords with little or no research significance were excluded, and those representatives of the research trends on VTE were focused. Aspirin ranked the highest burst strength (21.40), followed by surgery (21.29), and venous thrombosis (20.51). The latest burst keywords included aspirin, rivaroxaban, risk, complication, and total joint arthroplasty.

## Discussion

In this study, a bibliometric analysis was performed to visualize the results of global research status on VTE after hip or knee arthroplasty from 1990 to 2021, to reveal changes in research hotspots, and to predict future research trends. Publications on VTE have been numerous and increasing in the past decades (Figure 2). These articles originated from 53 countries and 603 institutions. Accordingly, we think that VTE is a growing research theme and an increasingly important research area worldwide. The USA was the dominant country with the largest number of articles (Table 1). In addition, half of the top 10 most prolific institutions were located in the USA, as shown in Table 2. Besides, in the top 10 most prolific authors (Table 3), four of them were from American institutions. Of note, the two most prolific and most-cited authors were Eriksson BI from Sweden and Lassen MR from Denmark, respectively. The co-cited author network (Figure 3D) showed that the top five co-cited authors were Geerts WH, Eriksson BI, Turpie AGG, Lassen MR, and Hull RD. Authors with high citations and co-citations are generally considered to have made outstanding contributions to the field (14). For beginners, choosing to read these authors' articles would help them to quickly understand the research foundations. Scholars may also choose these institutions or authors for VTE research exchanges and collaborations.

The journals which published the largest number of articles in VTE are bellwethers in orthopedic journals and thrombosis-related journals (Table 4). This trend pointed out that VTE is one of the core topics of orthopedics and thrombosis research. These journals were most favored by researchers around the world, which implies their high reputations and authority within the field of VTE research. Studies published in these prestigious journals are more likely to be recognized and cited. The New England Journal and The Lancet both with high impact factors published the most frequently cited articles in this field by Eriksson BI, Lassen MR, Kakkar AK, and Turpie AGG (22–28). Given the wider influence of these journals in their fields to attract readers and citations, scholars will prefer to submit their high-quality works to these journals.

The article's level of evidence rating can be very helpful to orthopedic surgeons in clinical treatment decisions (20). This study found that ~24% of the articles were randomized controlled trials (RCTs) and were rated as level I evidence (Figure 4). In contrast, 36% of the articles were case-control studies and retrospective cohort studies and were rated as level III evidence. In this study, the level I evidence articles were mainly about VTE prophylaxis agents which require rigorous high-ranking evidence studies to clarify their efficacy and safety. Upon statistical analysis, the median number of citations for level I evidence was significantly higher than those for other levels of evidence, demonstrating that articles with higher levels of evidence are favored by VTE researchers and osteopathic physicians. However, the low positive correlation between citations and levels of evidence may be due to less accumulation of citations in articles published recently or the shift in research hotspots to risk factors with level III or IV evidence (29, 30).

Literature with high citation can be considered the most valuable and influential studies in a certain field, hence, new researchers in a particular field could read these papers first before conducting further studies (14, 31). Nine of the top 10 highly cited articles were RCTs with level I evidence, focusing on comparations between rivaroxaban (22–24, 28), dabigatran etexilat (25, 32), and apixaban (26, 27, 33) vs. enoxaparin, respectively (Table 5). These three oral agents were found to be more effective than enoxaparin in VTE Prophylaxis after TKA or THA, and there was no statistical difference in the risk of bleeding.

The analysis of co-cited reference clustering and keyword co-occurrence can reflect the research hotspots in the field. Co-cited reference clustering analysis is a process of simplifying co-cited articles to a few clusters based on co-cited networks. The timeline of co-cited reference clustering and keyword burst detection can further track the evolution of research hotspots and predict future research trends (14). In this study, the most frequent keywords included VTE, PE, DVT, arthroplasty, prevention, enoxaparin, low molecular weight heparin (LMWH), and risk factor, indicating that the management of VTE after arthroplasty was of considerable interest to researchers around the world (Figure 6A). In addition, sixteen co-cited article clusters were identified (Figure 5). The serial numbers were arranged according to the cluster size, and they divided the field into different topics in detail. A chronological summary of these clusters and bursting keywords revealed that research hotspots switched from VTE diagnosis (cluster #14) and heparin (cluster #3, #1, #9) to factor Xa inhibitors (fondaparinux, rivaroxaban, apixaban, cluster #0, #8) and direct thrombin inhibitors (dabigatran etexilate, ximelagatran, cluster #10), and finally to aspirin (cluster #6), risk factor studies (cluster #11), and complication (22, 24, 25, 27, 34–39). Some topics burst with long durations, such as VTE and LMWH with a time-span of both 15 years, and rivaroxaban lasting from 2010 until now (23, 35) (Figure 6B). Keyword bursts in the early 21st century showed that the scholars mainly focused on factor Xa inhibitors (fondaparinux, rivaroxaban, apixaban) and direct thrombin inhibitors (dabigatran etexilate, ximelagatran) (25, 27, 38). Among the newer burst keywords, aspirin was a hot topic with the highest strength of 21.4 from 2014 to 2021 (8, 39–41). The constant shift of research hotspots for VTE prophylaxis indicated that scholars were always seeking more affordable, better tolerated, safer, and more effective oral drugs.

We have to acknowledge that this study has several limitations. First, the databases are continuously updated and only the WOSCC database was analyzed in this study. Therefore, some articles published in other databases may be omitted. Second, similar words such as the abbreviated forms and the plural forms need to be merged during the analysis, which makes the process cumbersome. Finally, some important recent articles may not have insufficient time to accumulate citations, which leads to the fact that recent breakthroughs might not draw enough attention yet. A follow-up study could be conducted in the future to evaluate the influence of these articles in the field.

## Conclusions

To our knowledge, this report is the first bibliometric analysis to provide a novel insight into the global evolving research foci and trends on VTE after hip or knee arthroplasty. Our findings suggest that the management of VTE post-arthroplasty has generated a tremendous and increasing research interest over the past few decades, ultimately exerting a critical influence on clinical decision-making. The largest contributions were made by the USA, and we found that the higher level of evidence of a publication got, the more often it would be cited. Particular areas of high research attention included VTE prophylaxis agents (warfarin, LMWH, direct factor Xa inhibitors, direct thrombin inhibitors, and aspirin) and risk factors, based on which keyword trends predict that future research may focus on individualized VTE prevention. Furthermore, new, safe, effective, and inexpensive oral agents need to be further developed. In summary, this study provides a comprehensive analysis of global research status and hotspots, and thus lays the groundwork for future research.

## Data Availability Statement

The original contributions presented in the study are included in the article/supplementary material, further inquiries can be directed to the corresponding authors.

## Author Contributions

WS and TM: writing-original draft. PW: conceptualization, project administration, and writing-review and editing. WS, TM, QC, and JW: data curation and methodology. LH and BZ: formal analysis and validation. YW and QW: resources and software. All authors have read and approved the submitted version.

## Funding

This work was supported by the Youth Cultivation Project of Xi'an Health Commission (Program No. 2020qn18) and the Key Research and Development Program of Shaanxi Province (Program No.2022SF-237).

## Conflict of Interest

The authors declare that the research was conducted in the absence of any commercial or financial relationships that could be construed as a potential conflict of interest.

## Publisher's Note

All claims expressed in this article are solely those of the authors and do not necessarily represent those of their affiliated organizations, or those of the publisher, the editors and the reviewers. Any product that may be evaluated in this article, or claim that may be made by its manufacturer, is not guaranteed or endorsed by the publisher.
